# A survey of senior medical students’ attitudes and awareness toward teaching and participation in a formal clinical teaching elective: a Canadian perspective

**DOI:** 10.1080/10872981.2016.1270022

**Published:** 2017-01-06

**Authors:** J. D. Matthew Hughes, Elise Azzi, Gregory Walter Rose, Christopher J. Ramnanan, Karima Khamisa

**Affiliations:** ^a^ Medical Student, Faculty of Medicine, University of Ottawa, Ottawa, Canada; ^b^ Department of Family Medicine, Faculty of Medicine, University of Ottawa, Ottawa, Canada; ^c^ Department of Medicine, Faculty of Medicine, University of Ottawa, Ottawa, Canada; ^d^ Department of Innovation in Medical Education, Division of Clinical and Functional Anatomy, Faculty of Medicine, University of Ottawa, Ottawa, Canada; ^e^ The Ottawa Hospital, General Campus, Ottawa, Ontario, Canada

**Keywords:** Curriculum, clinical teaching, electives, undergraduate medical education, near-peer teaching

## Abstract

**Background**: To prepare for careers in medicine, medical trainees must develop clinical teaching skills. It is unclear if Canadian medical students need or want to develop such skills. We sought to assess Canadian students’ perceptions of clinical teaching, and their desire to pursue clinical teaching skills development via a clinical teaching elective (CTE) in their final year of medical school.

**Methods**: We designed a descriptive cross-sectional study of Canadian senior medical students, using an online survey to gauge teaching experience, career goals, perceived areas of confidence, and interest in a CTE.

**Results**: Students at 13 of 17 Canadian medical schools were invited to participate in the survey (4154 students). We collected 321 responses (7.8%). Most (75%) respondents expressed confidence in giving presentations, but fewer were confident providing bedside teaching (47%), teaching sensitive issues (42%), and presenting at journal clubs (42%). A total of 240 respondents (75%) expressed interest in participating in a CTE. The majority (61%) favored a two week elective, and preferred topics included bedside teaching (85%), teaching physical examination skills (71%), moderation of small group learning (63%), and mentorship in medicine (60%).

**Conclusion**: Our study demonstrates that a large number of Canadian medical students are interested in teaching in a clinical setting, but lack confidence in skills specific to clinical teaching. Our respondents signaled interest in participating in an elective in clinical teaching, particularly if it is offered in a two-week format.

## Introduction

To prepare for careers in medicine, medical students must develop clinical teaching skills [1–3]. As residents, medical graduates are typically active in educator roles and are responsible for a considerable component of the medical education of clinical clerkship students [2]. In principle, medical students who teach become better learners, more effective communicators and better prepared to teach later in their careers [3]. This implies a benefit from early involvement and engagement in clinical teaching.

Many medical schools provide opportunities for medical students to develop scholarly research skills as prescribed by formal medical school accreditation standards [4,5]. On the other hand, there are no formal accreditation requirements mandating the promotion of teaching skills to undergraduate medical students. In 2008, a survey of 130 medical programs in the United States indicated that 44% (43 of 99 respondents) offered student-as-teacher (SAT) programs [6]. Prevailing content within these programs includes clinical teaching skill development, small group teaching skills and feedback derived from direct observation or videotaped teaching activity [6]. However, this study also indicated that a number of barriers exist regarding these SAT programs, including the challenge of creating a sense of value attributed to these initiatives, the lack of established national teaching competency standards to guide these programs, and the challenge of collating objective longitudinal data for the purposes of program evaluation [6]. Nonetheless, at least in the United States, many medical schools appear to provide formal clinical teaching skill training for their medical students. As of 2015, at least in the literature, it appeared that no Canadian medical school program offered the opportunity to develop clinical teaching skills to their medical students.

Medical residents and clinicians teach in multiple settings. However, over the past twenty years, bedside teaching has diminished as a teaching modality with only 17% of students receiving dedicated bedside teaching [7]. In 2015, to help address the deficiency of clinical teaching skills training, the University of Ottawa Faculty of Medicine established Canada’s first clinical teaching elective (CTE) [8]. This was offered to medical students in their final year of undergraduate medical education. The elective was intended for a small number of fourth year medical students [5,6]; however, a surprisingly high number of students sought enrollment in the elective, with 25–30 students (out a class of 150) registering and eventually participating in the CTE [8]. It was unclear whether this interest in clinical teaching was unique to our institution or whether there was a greater sense of interest in the broader population of senior medical students.

There is currently little known regarding Canadian medical student perceptions of clinical teaching training at the undergraduate level, including motivations for pursuing such training, barriers to participation and what objectives and content should be included in such initiatives. As such, the primary aim of this study was to conduct a national needs assessment, based on medical student self-perceptions to guide the development of a formal elective in clinical teaching. This needs assessment study would address perceived areas of deficiency in clinical teaching skills in senior medical school students, and would aim to design a clinical teaching skills initiative that conveys the teaching skill competencies required by Canadian medical school students.

## Methods

### Respondents and setting

Most Canadian medical schools offer a four-year Doctor of Medicine (MD) program, with two years of pre-clinical education followed by two years of clinical clerkship. Two Canadian medical schools (McMaster University and the University of Calgary) have a three-year MD program, with clinical clerkship occurring in the second and third years. Three of 17 medical schools in Canada offer instruction exclusively in French (these schools are in the province of Quebec). The survey was open to all senior medical students (in their last two years of training).

#### Ethics

Ottawa Health Science Network Research Ethics Board granted ethics approval for our study.

#### Survey

We developed a 15-question online survey, available in English and French on the Survey Monkey platform (see Table 1). We asked basic demographic characteristics; dichotomous (‘yes/no’) questions regarding teaching or research experience and future plans; and questions regarding confidence in their own teaching abilities rated on a 5-point Likert scale (‘strongly disagree’ to ‘strongly agree’). For each quantitative survey item, the option to provide open-ended qualitative feedback (if students felt the need to expand or justify their Likert selection) was provided via a comment box. The survey was developed with two medical education consultants and piloted by three medical students prior to distribution.

**Table 1.** English survey questions.

Question #	Question
**1**	State your age
**2**	Select your gender
**3**	Select the Faculty of Medicine to which you are affiliated
**4**	Please indicate your intended residency choice
**5**	Do you have any prior teaching experience?
**6**	If yes, please select all those that apply (e.g. Lecturer, Peer tutor, TAClinical teacher, Other)
**7**	Do you plan on working in an academic hospital?
**8**	Are you interested in pursuing clinical teaching for medical students/residents upon completion of residency?
**9**	If yes, please indicate which factors motivate you to engage in clinical teaching (e.g. Academic Advancement, Intrinsic Interest, Prestige, Requirement to work in an academic centre, Desire to ‘give back’, Increase confidence in teaching, Other)
**10**	Have you undertaken research in the area of medical education?
**11**	Confidence in Teaching abilities (Likert scale)
**12**	I would participate in a clinical teaching elective during my last year of medical school (Likert scale)
**13**	What would the ideal duration for the elective be?
**14**	What topics would you include in a two-week medical education/clinical teaching elective?
**15**	If you have any other comments, please enter them below

TA: teaching assistant.

#### Recruitment and survey distribution

We recruited respondents indirectly through their national student representatives. Briefly, each Canadian medical school has student representation on the Canadian Federation of Medical Students (CFMS). These representatives were contacted via email and these students agreed to disseminate the survey to the target medical student population at their home institution (note, each student was not contacted directly by our researchers). The student representatives then relayed these emails (including a brief introduction to the survey in English and in French, as well as the link to access the survey) to the senior medical students at each school, with one reminder email sent two weeks before the survey was closed. In total, 13 CFMS representatives (out of 17) agreed to help disseminate this survey to their local institutions. As such, the data collected from our survey represents the views from 76% of Canadian medical schools. At the time of the study there were 5583 senior medical students registered in the 17 Canadian Faculties of Medicine [9]. A total of 4154 students had access to the survey (from the 13 participating medical schools) and there were 321 eventual respondents, resulting in a response rate of 7.7% of those surveyed.

#### Statistical analysis

We computed simple descriptive statistics (frequencies and percentages) for each survey question using Survey Monkey.

## Results

We collected 321 responses from senior medical students at 13 Canadian medical schools. Four Canadian medical schools declined to participate, including all three unilingual Francophone schools.

Demographic information is outlined in Table 2. Of the 321 respondents, 311 (96%) responded to the survey in English while 10 responded to the survey in French. The majority of respondents were female (69%).

**Table 2.** Demographic characteristics of 321 senior medical student survey respondents*

Characteristics	N (%)
Sex	
Male	98 (30.7%)
Female	220 (69%)
Prefer not to state	1 (0.3%)
Age	
≤ 18	0
19–24	160 (50%)
25–29	132 (41%)
30–34	22 (7%)
35–39	7 (2%)
≥ 40	0
Medical School	
University of Ottawa	71 (22%)
University of British Columbia	45 (14%)
University of Toronto	37 (12%)
Dalhousie University	34 (11%)
McMaster University	30 (9%)
McGill University	28 (9%)
University of Alberta	27 (8%)
University of Manitoba	15 (5%)
Queen’s University	10 (3%)
Western University	10 (3%)
Memorial University of Newfoundland	9 (3%)
University of Saskatchewan	3 (1%)
University of Calgary	2 (1%)

*Not all respondents answered all the questions.

The majority (74%) had prior teaching experience, of which the most common was as a tutor/peer tutor/supplemental instructor (either in premedical or early medical school training) as indicated in Table 3. Many survey respondents have academic plans: 95% will pursue clinical teaching upon completion of their residency, and 71% would like to work in an academic hospital. 53% of respondents hope to train in one of three disciplines: family medicine (27%), internal medicine (16%) and pediatrics (10%)

**Table 3.** Teaching experience and future career goals of 321 senior medical student survey respondents

Survey question	Response
‘Do you have any prior teaching experience?’	
Yes (%)	74
No (%)	26
Total (n)	320
‘Have you undertaken research in the area of medical education?’	
Yes (%)	28
No (%)	72
Total (n)	321
‘Do you plan on working in an academic hospital?’	
Yes (%)	71
No (%)	29
Total (n)	317
‘Are you interested in pursuing clinical teaching for medical students/residents upon completion of residency?’	
Yes (%)	95
No (%)	5
Total (n)	321
‘Please indicate your intended residency choice’ (N = 314)	
Family Medicine (n)	86
Internal Medicine (n)	50
Pediatrics (n)	32
Emergency Medicine (n)	23
Obstetrics and Gynecology (n)	18
Anesthesiology (n)	8
General Surgery (n)	7
Diagnostic Radiology (n)	7
Ophthalmology (n)	7
Plastic Surgery (n)	7
Neurology (n)	6
Prefer not to state (n)	27
Other (n)	36

Many respondents felt confident giving presentations (75% agreeing or strongly agreeing) and facilitating small group sessions (71%), while fewer felt confident providing bedside teaching (47%), teaching sensitive issues (42%), and presenting at journal clubs (42%) (Table 4). The majority of students felt confident providing written feedback to learners (74%) and 67% felt confident in providing verbal feedback.

**Table 4.** Perceived confidence in teaching abilities expressed by 319 senior medical student survey respondents.

Answer Options	Strongly disagree	Disagree	Neutral	Agree	Strongly agree
I feel confident giving presentations.	2%	5%	18%	**56%**	**19%**
I feel confident facilitating small group sessions.	1%	8%	20%	**52%**	**18%**
I feel confident performing bedside teaching.	3%	17%	34%	**38%**	**8%**
I feel confident teaching about sensitive issues, communication, and ethics.	3%	23%	32%	**33%**	**9%**
I feel confident presenting at a journal club.	4%	26%	28%	**32%**	**10%**
I feel confident giving verbal feedback.	2%	8%	23%	**56%**	**11%**
I feel confident giving written feedback.	1%	7%	18%	**53%**	**21%**

**Figure 1.**
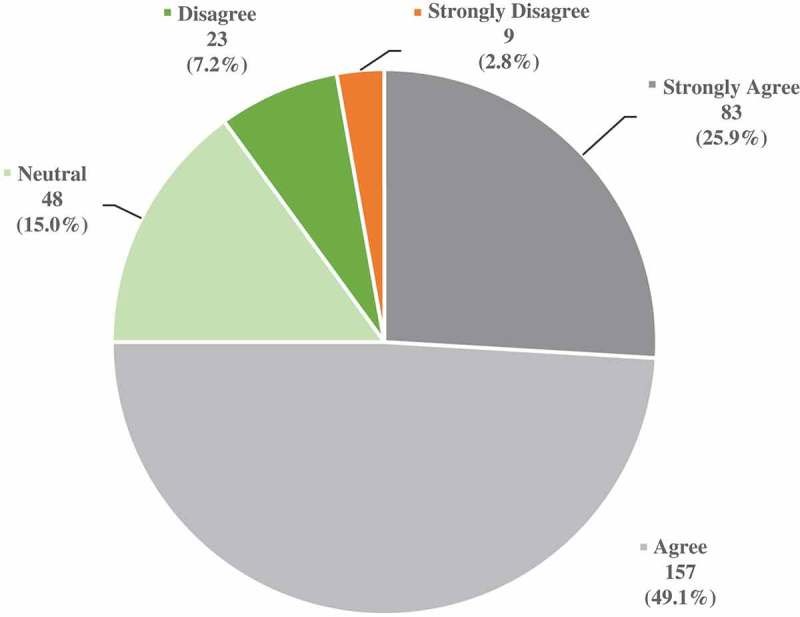
Response of 320 senior medical student survey respondents to statement ‘I would participate in a clinical teaching elective during my last year of medical school’.

Of the students surveyed, the majority (75%) agreed/strongly agreed that they would like to participate in such a clinical teaching elective, while 15% were neutral and 10% disagreed/strongly disagreed (Figure 1). In follow-up questioning, 61% of respondents indicated two weeks would be the ideal duration for the elective, while 31% preferred one week, and 4% chose one month.

The needs analysis (Table 5) showed that students would like sessions on the following topics: bedside teaching (85%), teaching physical examination skills (71%), moderation of small group learning (63%), and how to mentor in medicine (60%).

**Table 5.** Clinical teaching elective topics preferred by 318 senior medical student survey respondents*

Topics	%
Bedside teaching	85
Teaching physical exam skills	71
Effective moderation of small group learning	63
Mentorship in medicine	60
Effective presentation skills	54
Simulation in medical education	51
Leadership in medicine	47
Assessment in medical education	43
Teaching in the ambulatory care setting	43
Teaching in the emergency room setting	43
How to give effective journal club presentations	38
Curriculum design in medical education	37
How to publish in the field of medical education	24

*Respondents were able to select more than one topic

## Discussion

Our study demonstrates that the majority of senior Canadian medical student survey respondents are interested in, but not prepared for, teaching in a clinical setting. While 74% of survey respondents reported some form of teaching experience, only 3.3% of those had experience as clinical teachers (Table 3). A total of 95% planned to deliver clinical teaching in their career, but only 47% felt confident with bedside teaching (Table 4); only 42% of student were comfortable conducting and presenting at journal club, an expected educational task in most residency programs [10].

The second objective of the study was to assess student interest in participating in a clinical teaching elective if offered at their institution (Figure 1); 75% of survey respondents expressed interest in such an elective, with the majority indicating an ideal duration of two weeks.

This is the first Canadian study to ask these questions of medical students. In Australia, Burgess and colleagues evaluated the impact of a similar educational program, the ‘Teaching on the Run’ (TOR) program [11]. Prior to participating in TOR, Year three medical students expressed lack of confidence in their understanding of key educational principles and activities. Also, students were very interested in TOR – 67% of eligible students participated in the program. A key study by Soriano *et al* also noted that close to 50% of all U.S. medical schools offer students as teachers (SAT) training with 31% of schools offering two or four week teaching electives [6].

In this study, students were also surveyed regarding potential curricular content of a clinical teaching elective. Despite the advances in curriculum development and delivery over the past 10 years, students still have an overwhelming desire to be good bedside teachers and acquire confidence in explaining physical examination maneuvers to junior colleagues (Table 5). 85% of respondents felt bedside teaching to be an important component of such an elective, while students were least interested in including topics pertaining to publishing in the field of medical education (24%). The present clinical teaching elective offered at our institution uses standardized patients and small group sessions to help students improve bedside teaching techniques [8]. The evaluation component of this elective is currently in progress, but it is interesting to note other studies showing a potential long-term impact of such electives on clinical teaching outcomes [12].

Our study has two major limitations, first of which is the low response rate. While 321 senior medical students completed our survey, there are approximately 5583 senior medical students in Canada this year. The survey was advertised indirectly to 4154 students from 13 of 17 medical schools. The relatively low participation rate in this population has historically been attributed to survey fatigue and lack of time in clinical clerkship settings. We were unable to contact each student directly to participate in the survey, but relied upon school representatives to forward on the survey link which may have had reduced efficiency in the delivery of the survey. Despite this, there would likely be a reasonable absolute number of students who would engage in a formal teaching elective if offered at each medical school. Even if we were to presume that our respondents were the only students in Canada interested in participating in a clinical teaching elective, this is still potentially 240 students – enough for an 8–10 person elective at each academic year at each participating school. During the final year of medical training, elective time in Canadian medical schools is quite limited and often geared towards electives that will enhance the chances of matching to limited residency positions. Hence, it is interesting to note that if given the chance, many students would participate in such an elective, which is not directly linked to any particular residency program. Based on our small study, the inclusion of such an elective should strongly be considered as a curricular component in the last year of medical training.

A further limitation of our study is the existence of selection bias. First, our inability to survey students at four of 17 Canadian medical schools could be problematic. It is conceivable that students at the non-participating schools (a distributed medical education school, and three unilingual Francophone schools) may have less interest in clinical teaching than students at the 14 participating schools, but we believe this is unlikely.

Additionally, there were a high number of respondents interested in matching to residency positions in family medicine, internal medicine, and pediatrics; it is plausible that students interested in these specialties are more interested in clinical teaching than other students are. These three specialties account for the bulk of clinical learning experience for medical students, so it would be natural for students to associate them with opportunities for clinical teaching. Pragmatically this does not detract from our findings. Indeed, our sample is reflective of the Canadian reality: 53% of our respondents indicated an interest in one of these three specialties, whereas post-graduate training data indicate 65% of graduating medical students will start a residency in one of these specialties [5].

## Conclusion

Our study demonstrates a large number of senior medical students at Canadian medical schools are interested in developing their clinical teaching abilities, with a particular interest in participating in a 2-week elective during the latter part of medical school training. Based on our data, a key component of such an elective would be teaching medical students to be confident bedside teachers.
